# The prebiotic effects of soluble dietary fiber mixture on renal anemia and the gut microbiota in end-stage renal disease patients on maintenance hemodialysis: a prospective, randomized, placebo-controlled study

**DOI:** 10.1186/s12967-022-03812-x

**Published:** 2022-12-14

**Authors:** Yang Li, Min Han, Jia Song, Shijin Liu, Yongjun Wang, Xinhuan Su, Kai Wei, Zhen Xu, Hui Li, Zunsong Wang

**Affiliations:** 1 Department of Nephrology, The First Affiliated Hospital of Shandong First Medical University & Shandong Provincial Qianfoshan Hospital, Shandong Institute of Nephrology, No. 16766 Jingshi Road, Jinan, 250014 Shandong China; 2 Shandong First Medical University, No. 6699 Qingdao Street, Jinan, 250117 Shandong China; 3 Weifang Medical University, No. 7166 Baotong West Street, Weifang, 261053 Shandong China; 4 Department of Clinical Nutrition, The First Affiliated Hospital of Shandong First Medical University & Shandong Provincial Qianfoshan Hospital, No. 16766 Jingshi Road, Jinan, 250014 Shandong China; 5 Department of Endocrinology, Shandong Provincial Hospital Affiliated to Shandong First Medical University, Jinan, 250021 Shandong China; 6 Department of Nephrology, Yuncheng Chengxin Hospital, West of Jiangmiaodeng Tower, Yunzhou Street, Heze, 274700 Shandong China; 7 Department of Nephrology, People’s Hospital of Lingcheng, No. 245 Zhongxing Road, Dezhou, 253599 Shandong China

**Keywords:** Renal anemia, Dietary fiber, Gut microbiota, Hemodialysis, End-stage renal disease, Short chain fatty acids

## Abstract

**Background:**

Renal anemia is caused by end-stage renal disease (ESRD) but has a complex etiology. The application of dietary fiber (DF) to regulate the gut microbiota has shown effective therapeutic effects in some diseases, but its role in renal anemia is not clear. The aim of this study was to explore the effect of DF on renal anemia by regulating the gut microbiota and its metabolite, short-chain fatty acids (SCFAs).

**Methods:**

A total of 162 ESRD patients were enrolled and randomly distributed into a DF or a control group (received oral DF or potato starch, 10 g/day for 8 weeks). Hemoglobin (Hb), serum iron (Fe^2+^), serum ferritin (SF), soluble transferrin receptor (sTfR), hepcidin and the dosage of recombinant human erythropoietin (rhEPO) before and after intervention in patients were analyzed. The gut microbiota and SCFAs in both groups were analyzed by 16S rDNA sequencing and gas chromatography–mass spectrometry, respectively. Spearman’s correlation test was used to analyze the correlation between the gut microbiota, SCFAs and the hematological indicators.

**Results:**

Compared with the control group, (1) the patients in the DF group had higher Hb [117.0 (12.5) g/L vs. 94.0 (14.5) g/L, *p* < 0.001], Fe^2+^ [13.23 (4.83) μmol/L vs. 10.26 (5.55) μmol/L, *p* < 0.001], and SF levels [54.15 (86.66) ng/ml vs. 41.48 (36.60) ng/ml, *p* = 0.003]. (2) The rhEPO dosage in the DF group was not significantly decreased (*p* = 0.12). (3) *Bifidobacterium adolescentis*, *Lactobacillus* and *Lactobacillaceae* were increased in the DF group, and *Lactobacillus* and *Lactobacillaceae* were positively correlated with Hb (*r* = 0.44, *p* < 0.001; *r* = 0.44, *p* < 0.001) and Fe^2+^ levels (*r* = 0.26, *p* = 0.016; *r* = 0.26, *p* = 0.016) and negatively correlated with rhEPO dosage (*r* = − 0.45, *p* < 0.001; *r* = − 0.45, *p* < 0.001). (4) Patients in the DF group had elevated serum butyric acid (BA) levels [0.80 (1.65) vs. 0.05 (0.04), *p* < 0.001] and BA levels were positively correlated with Hb (*r* = 0.26, *p* = 0.019) and Fe^2+^ (*r* = 0.31, *p* = 0.005) and negatively correlated with rhEPO dosage (*r* = − 0.36, *p* = 0.001). *Lactobacillus* and *Lactobacillaceae* were positively correlated with BA levels (*r* = 0.78, *p* < 0.001; *r* = 0.78, *p* < 0.001).

**Conclusion:**

DF may improve renal anemia in ESRD patients by regulating the gut microbiota and SCFAs.

*Trial registration* This study was registered in the China Clinical Trial Registry (www.chictr.org.cn) on December 20, 2018 (ChiCTR1800020232).

**Supplementary Information:**

The online version contains supplementary material available at 10.1186/s12967-022-03812-x.

## Introduction

Anemia is one of the common and serious complications of end-stage renal disease (ESRD), and it affects approximately 41% of ESRD patients [1]. Renal anemia is one of the important factors for heart failure and mortality in ESRD, which seriously affect the quality of life of ESRD patients [2]. Dietary fiber (DF) has been shown to improve anemia at the clinical and animal model levels. Carvalho et al. found that partially hydrolyzed guar gum (PHGG), a DF, was able to ameliorate anemia in rats by improving iron metabolism [3]. Additionally, Paganini et al. found that galactooligosaccharides (GOS), one type of DF, were able to improve iron absorption and improve anemia in humans [4]. Recently, a systematic review by Thaísa et al. showed that although there is heterogeneity in the conclusions of numerous current basic and clinical studies on DF for the improvement of anemia associated with iron metabolism, its potential role in the improvement of anemia and intestinal biomarkers should be emphasized [5]. Thus, further studies in this area are still needed.

DF is a polysaccharide derived mainly from plant-based foods that cannot be directly broken down and utilized by the human digestive system. Howerer, DF can serve as a fermentation substrate for gut microbiota residents [6, 7]. DF is divided into soluble DF and insoluble DF. Insoluble DF cannot be dissolved in water, and its main function is to store water in the colon, soften feces, and enhance bowel motility. Thus, DF has a certain therapeutic effect on constipation [8, 9]. Soluble DF is a type of DF that can dissolve in water; these include inulin, oligofructose, resistant starch, guar gum, etc. Humans do not have the genes to degrade polysaccharides, but bacteria can break them down. Therefore, fiber can be utilized by the human body as fermentation products, i.e., short-chain fatty acids (SCFAs). Some bacteria in the human gut are able to use DF as a catabolic substrate to produce substances such as SCFAs and bile acids, which is also one of the reasons why soluble DFs are considered to have prebiotic effects. Studies have reported that decreased SCFA levels in feces and/or serum are associated with a variety of diseases, including anemic disorders [10, 11].

There is heterogeneity in the results of some studies investigating the effects of DF on the treatment of anemia. These differences may be due to differences in different DF species but also by individual gene variability. Differences in the availability of resistant starch due to individual amylase gene heterogeneity were recently described by Dobranowski et al. [12]. However, because DF is not directly utilized by the human body, its catabolism is dependent on certain gut microbiota, such as *Akkermansia muciniphila* and *Clostridium butyricum*. Therefore, differences in the gut microbiota may also be one of the reasons for the different effects of DF. Deehan et al. found that arabinoxylan can be fermented by *Dialister invisus* and *Bacteroides plebeius* to produce propionic acid to improve insulin resistance, while the bile acid produced by the bacteria can improve intestinal inflammation [13]. Comparatively, DF also influences the gut microbiota and is able to remodel and ameliorate the disordered gut microbiota. Additionally, the increase in beneficial bacteria may in turn better utilize DF to produce beneficial substances such as SCFA. Ranaivo et al. showed that DF is able to modulate the gut microbiota structure and improve its metabolic profile in patients at risk for heart disease [14]. Moreover, an ameliorative effect of a composite DF diet on metabolic diseases was described by Cani et al. [15]. The effect of DF on whole-body metabolism is achieved by the gut microbiota, which metabolizes DF into substances such as SCFA. Xu et al. found that acetate could directly stimulate erythropoiesis through a hypoxia inducible factor-2 (HIF-2)-related pathway [16]. A study by Reid et al. found that butyric acid (BA) derivatives ameliorated sickle cell anemia [17]. Weinberg et al. reported that BA was able to promote the synthesis of γ-globin [18]. However, a study by Soriano-Lerma et al. found that propionate, BA and their related producing bacteria are effective against iron deficiency anemia [10]. The above studies indicate that SCFAs and the gut microbiota are able to affect anemia-related diseases through multiple pathways and are worthy of further study.

The aim of this study was to explore the role of DF, the gut microbiota and SCFAs in renal anemia and to preliminarily explore the possible mechanism underlying this effect using a combination of genomics and metabolomics. We hypothesized that adequate daily intake of DF would improve renal anemia in Chinese participants by modulating the gut microbiota and increasing the concentration of SCFAs. This study explores the role of DF in ameliorating renal anemia. Moreover, this study links DF, the gut microbiota, SCFA, and anemia in an attempt to provide new insights into this regimen of DF modulation of the gut microbiota to ameliorate renal anemia.

## Participants and methods

### Participants

A total of 384 ESRD maintenance hemodialysis patients with renal anemia were recruited from January 1, 2019, to December 31, 2019, at three different dialysis centers in Shandong Province, China (Shandong Provincial Qianfoshan Hospital, People's Hospital of Lingcheng District of Dezhou, and Yuncheng Chengxin Hospital of Heze). Ultinately, 162 patients were enrolled in the study. The inclusion criteria were as follows: (1) received maintenance hemodialysis for ≥ 3 months; (2) 18–80 years old; (3) stable condition; (4) hemoglobin(Hb) < 110 g/L; (5) full civil competence; and (6) able to understand and agree to the content of this study and signed informed consent. The exclusion criteria were as follows: (1) severe anemia (Hb < 60 g/L); (2) comorbid viral hepatitis, tuberculosis, sexually transmitted diseases and other infectious diseases; (3) combined solid tumors or hematologic tumors; (4) infection; (5) gastrointestinal disorders such as diarrhea, constipation, and dyspepsia; (6) pregnancy or lactation; (7) any dosage form/dose of antibiotic had been applied within 8 weeks prior to enrollment; (8) serum ferritin concentration (SF) > 200 ng/mL; and (9) received intravenous or oral iron supplement. All patients were randomly assigned to the DF intervention group and placebo control group (potato starch).

The primary outcome of this study was the change in Hb, and the secondary outcomes were the change in iron metabolism-related indices, SCFAs, gut microbiota composition and the dosage of exogenous recombinant human erythropoietin (rhEPO).

In particular, we were unable to determine an appropriate sample size calculation due to a lack of published similar studies to date. Thus, we enrolled as many participants as feasibly possible.

### Methods

#### Baseline characteristics

1. Basic data, such as the patient’s name, sex at birth, age, estimated glomerular filtration rate (eGFR), body weight, time on hemodialysis and primary disease were collected and recorded.
2. Hb concentration, serum ferric ion concentration (Fe^2+^), SF, and soluble transferrin receptor concentration (sTfR) at the last laboratory examination before patient enrollment were recorded, and rhEPO dosage (calculated as the average dosage over the 4 weeks before enrollment) was recorded.

#### Intervention protocol

1. Each enrolled patient was provided with equipment, such as a food scale and oil and salt graduated cylinder/spoon free of charge, and the patients were taught food classification and weighing methods by trained dedicated personnel. Three-day dietary records were used to record the type and quantity of diet the patients consumed on three consecutive days. Their daily habits, including all food intake such as drinking water and snacks, were recorded. The daily DF intake of each patient was analyzed by using Feihua Nutrition Calculation Software (version 2.7.8.4). The final results were calculated as 3-day averages.
2. According to the survey results and relevant guidelines [19–21], the DF intervention group patients received a 10 g DF mixture (prepacked powder) orally every day (purchased from Shanghai Xianbo Food Co., Ltd., mainly composed of galactomannan, resistant dextrin, fructooligosaccharide and starch). The control group was given an oral dose of 10 g/day potato starch (derived from potato and prepacked with the same dosage and packing bag as the DF mixture, except for the color of bag). The intervention time of both groups was 8 weeks (56 days).

#### Sample collection and measurement

1. Collection and detection of blood samples
  Blood samples were collected by dedicated nursing staff. Hb, Fe^2+^, SF, and sTfR were measured by the department of laboratory medicine at each center. The departments of each dialysis center have passed the provincial-wide unified quality inspection, and the detection results are consistent.
  The freshly collected blood was allowed to stand for 30 min at room temperature, and the supernatant (serum) was collected after centrifugation at 3600 rpm for 10 min at room temperature. Then, they were stored at − 80 ℃ and transported on dry ice. The serum concentration of hepcidin was detected by ELISA (Elabscience Biotechnology Co., Ltd., Wuhan, China). The measurement of SCFAs, including acetic acid (AA), propionic acid (PA), butyric acid (PA), isobutyric acid (IBA), valeric acid (VA), isovaleric acid (IVA) and hexanoic acid (HA), was completed by Wuhan MetWare Biotechnology Co., Ltd. using the gas chromatography–mass spectrometry (GC–MS) method.
2. Collection and detection of fecal samples
  After the 8-week intervention period, all patient feces samples were collected by applying a sterile stool collector, and the samples were stored at − 80 ℃ for 16S rDNA sequencing. 16S rDNA sequencing was performed by Hangzhou Lianchuan Biotechnology Co., Ltd. The details are shown in Additional file 1.

#### Statistical analysis

According to the data type, the measurement data are expressed as the mean ± standard deviation (${\overline{\text{x}}}$ ± SD) or median (interquartile range) [M (IQR)], and the counting data are expressed by relative number. Comparisons of clinical data, SCFA and relative abundance of *Bifidobacterium adolescentis*, *Lactobacillus* and *Lactobacillaceae* between the DF and control groups were performed with independent sample *t* tests, Mann–Whitney *U* tests or chi-square tests according to the characteristics of the data distribution. Detailed methods for 16S rDNA sequencing are presented in Additional file 1. *p* < 0.05 was considered statistically significant. The statistical analysis was completed by SPSS software (version 26.0, IBM, USA).

## Results

### Patient recruitment

**Fig. 1:**
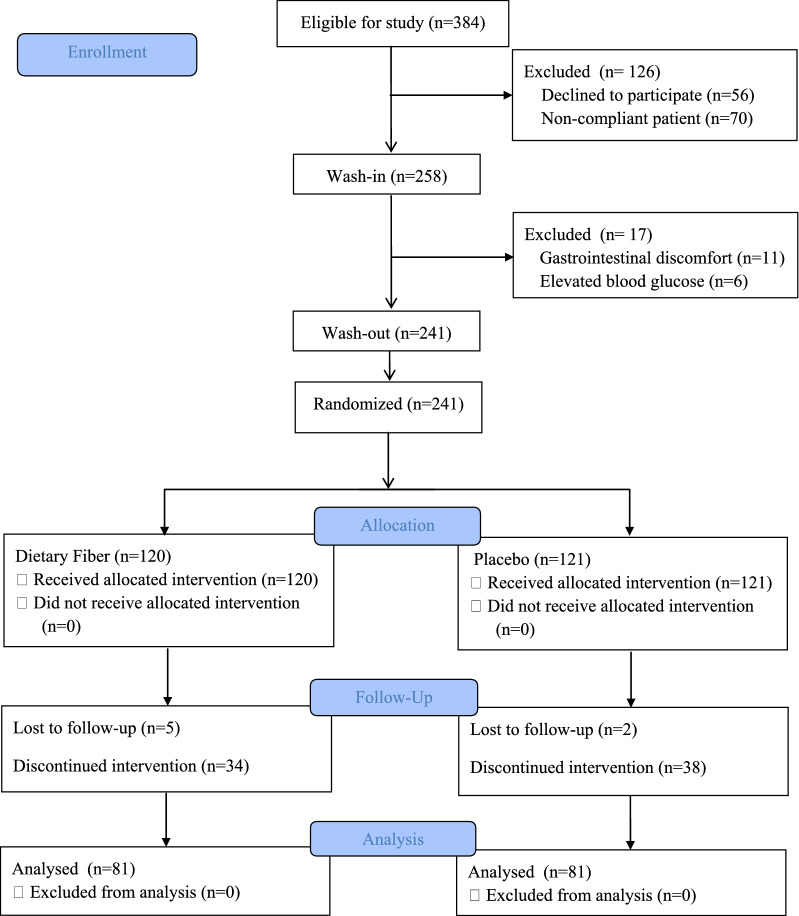
CONSORT diagram: flow of study participants from screening to study completion

Initially, 384 patients who met the criteria were included in this study. After the initial review, 126 patients were excluded, including 56 patients who refused to participate in the study and 70 patients who were considered to be poorly adherent based on their weekday performance, such as inability to tightly control their diet or inability to take their medication on time. After initial screening, 258 patients entered the wash-in period. During this period, patients were asked to consume 10 g of DF orally daily for 2 weeks. After this period, 17 patients were excluded, including 8 patients who presented with abdominal distension, 2 patients who presented with diarrhea, 1 patient who presented with constipation, and 6 patients who presented with an increase in blood glucose levels of more than 1 mmol/L from baseline. Finally 241 patients entered the wash-out period. During this period, patients were asked to eat a normal diet and to stop taking additional DF supplements for 2 weeks, with the goal of eliminating the effects from DF taken during the wash-in period. After the wash-out period, 241 patients were randomized into a DF group (*n* = 120) and a control group (*n* = 121). During the 8-week intervention period, 1 patient in the DF group died in a car accident, 4 patients withdrew from the study with the intention of receiving a kidney transplant, and 34 patients were excluded from the study because they did not take the DF on time during the follow-up period. Moreover, 2 patients in the control group died of acute cardiovascular disease, and 38 patients were excluded from the study because they did not take the DF on time during the course of the follow-up period. Ultimately, 81 patients in each of the two groups completed all interventions and entered the analysis phase. The results are shown in Fig. 1.

### Baseline characteristics of patients

A total of 162 enrolled patients were randomly divided into a DF group and a control group. There were no significant differences in sex at birth, age, primary disease, Hb, Fe^2+^, SF, sTfR, hepcidin or rhEPO dosage among the patients in each group before intervention (Table 1).

**Table 1:** Baseline characteristics of final enrolled participants

	Total (*n* = 162)	Control group (*n* = 81)	DF group (*n* = 81)	*p* value
Sex at birth (*n*, %)				0.636
Male	87 (53.7%)	42 (51.9%)	45 (55.6%)	
Female	75 (46.3%)	39 (48.1%)	36 (44.4%)	
Age (years, ${\overline{\text{x}}}$ ± SD)	50.33 ± 8.31	49.62 ± 8.47	51.15 ± 8.13	0.274
Body weight (kg)	58.55 ± 10.32	59.86 ± 9.86	57.24 ± 10.66	0.120
Time in hemodialysis (months)	21.50 (28.50)	18.50 (37.50)	24.00 (24.00)	0.570
eGFR (mL/min/1.73 m^2^)	4.56 ± 2.60	4.51 ± 2.17	4.61 ± 3.10	0.936
Primary disease (*n*, %)				0.924
GN	72 (44.4%)	37 (45.7%)	35 (43.2%)	
DKD	50 (30.9%)	23 (28.4%)	27 (33.3%)	
HN	19 (11.7%)	10 (12.3%)	9 (11.1%)	
Others	21 (13.0%)	11 (13.6%)	10 (12.3%)	
Hb [g/L, M (IQR)]	93.00 (13.00)	93.00 (11.00)	94.00 (15.50)	0.231
Fe^2+^ [μmol/L, M (IQR)]	9.73 (4.33)	9.35 (4.77)	10.21 (4.20)	0.179
SF [ng/mL, M (IQR)]	31.78 (34.58)	33.43 (24.73)	30.40 (45.29)	0.758
sTfR [(g/L), M (IQR)]	1.50 (0.92)	1.36 (0.91)	1.63 (0.94)	0.140
Hepcidin [(pg/mL), M (IQR)]	54.14 (39.76)	51.09 (41.70)	56.69 (41.57)	0.253
rhEPO [IU/W, M (IQR)]	15,000.00 (5000.00)	15,000.00 (5000.00)	15,000.00 (5000.00)	0.830
DF intake (g/d)	10.07 ± 5.61	10.68 ± 6.35	9.41 ± 4.63	0.157

*GN* glomerulonephritis, *DKD* diabetic kidney disease, *HN* hypertensive nephropathy, *Hb* hemoglobin, *Fe*^*2*+^ serum iron, *SF* serum ferritin, *sTfR* soluble transferrin receptor, *rhEPO* recombinant human erythropoietin, *DF* dietary fiber, *eGFR* estimated glomerular filtration rate, *p value* DF group versus control group

### Comparison of hematological indices between the two groups after intervention

**Fig. 2:**
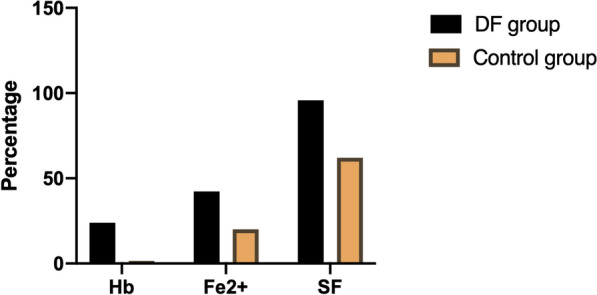
Bar chart of the percentage change in Hb, Fe^2+^, and SF at the end of 8 weeks compared with baseline for both the DF (n = 81) and control (n = 81) groups. *Hb* hemoglobin, *Fe*^*2*+^ serum iron, *SF* serum ferritin, *DF* dietary fiber

After the 8-week intervention, Hb, Fe^2+^, and SF were significantly higher in the patients in the DF group than in the patients in control group. The dosage of rhEPO in the DF group was not significantly different from that in the control group. There was no difference in sTfR or hepcidin between the two groups (Table 2). The percentage changes in Hb, Fe^2+^ and SF are shown in Fig. 2.

**Table 2:** Clinical indexes changes between groups after treatment period

	Control group (n = 81)	DF group (n = 81)	*p* value
Hb [(g/L, M (IQR)]	94.00 (14.50)	117.00 (12.50)	< 0.0001
Fe^2+^ [μmol/L,M (IQR)]	10.26 (5.55)	13.23 (4.83)	< 0.0001
SF [ng/mL, M (IQR)]	41.48 (36.60)	54.15 (86.66)	0.003
sTfR [(g/L), M (IQR)]	1.65 (0.75)	1.65 (0.88)	0.572
hepcidin [(pg/mL), M (IQR)]	58.85 (42.55)	58.11 (39.38)	0.988
rhEPO [IU/W, M (IQR)]	15,000.00 (6000.00)	12,000.00 (7000.00)	0.122

*sTfR* soluble transferrin receptor, *Hb* hemoglobin, *Fe*^*2*+^ serum iron, *SF* serum ferritin, *rhEPO* recombinant human erythropoietin, *IU/W* international unit per week, *DF* dietary fiber

### Comparison of the gut microbiota between the two groups after intervention

**Fig. 3:**
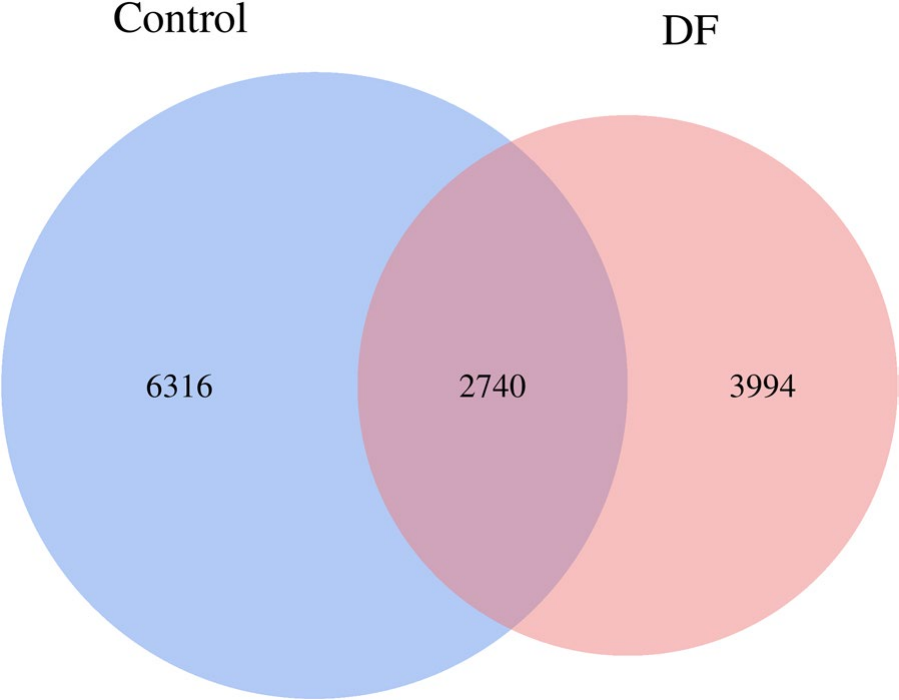
A Venn diagram of the control group and the DF group. There were 6316 unique bacteria in the control group and 3994 unique bacteria in the DF group, and 2740 bacteria were common to both groups. *DF* dietary fiber

**Fig. 4:**
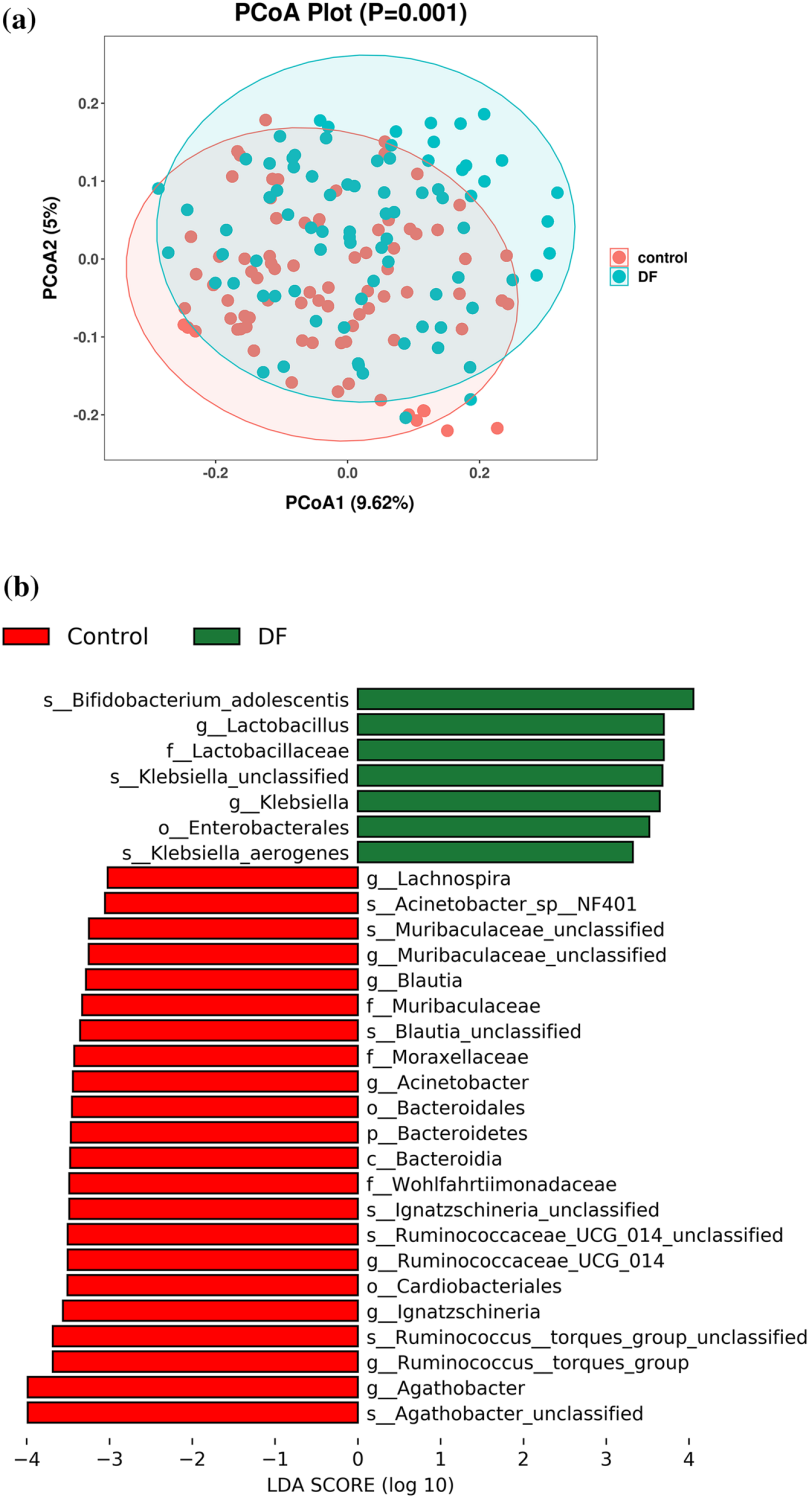
The differences in bacteria between the control group and the DF group. **a** PCoA based on unweighted UniFrac showing the differences in bacterial composition between the control group and DF group. **b** The major different bacteria between the control group and DF group were generated from LEfSe analysis (LDA score above 3.0)

After an 8-week intervention, the gut microbiota of patients in the DF group was significantly changed compared with that in patients in the control group. (1) Compared with those in the control group, patients in the DF group had more homogeneous species of the gut microbiota, and 3994 unique bacteria were identified (Fig. 3). (2) Compared with patients in the control group, the gut microbiota of patients in the DF group was changed at the phylum and genus levels, and the Firmicutes to Bacteroidetes ratio was decreased [177.68 (364.68) vs. 431.90 (576.70), *p* = 0.002]. These fidings are consistent with previous reports [22]. (3) Principal coordinate analysis (PCoA) revealed that the composition of the gut microbiota was different between patients in the DF group and those in the control group, and LEfSE (Linear Discriminant Analysis Effect Size) analysis revealed that *Bifidobacterium adolescentis*, *Lactobacillus* and *Lactobacillaceae* were increased in patients in the DF group (Fig. 4, Table 3, Additional file 2).

**Table 3:** Abundance results from between group comparisons of 3 targeted bacteria

Gut microbiota [%, M (IQR)]	Control group (*n* = 81)	DF group (*n* = 81)	*p* value
Bifidobacterium adolescentis	0.00 (0.04)	0.02 (0.09)	0.009
Lactobacillus	0.01 (0.06)	0.04 (0.15)	0.004
Lactobacillaceae	0.01 (0.06)	0.04 (0.15)	0.004

*DF* dietary fiber

### Serum SCFA changes in patients after intervention

Compared with the control group, the concentrations of BA [0.80 (1.65) vs. 0.05 (0.04), *p* < 0.0001], IBA [0.03 (0.06) vs. 0.02 (0.05), *p* = 0.002], VA [0.03 (0.04) vs. 0.02 (0.04)], IVA [0.03 (0.04) vs. 0.02 (0.04), *p* = 0.001] and HA [0.11 (0.03) vs. 0.07 (0.02), *p* < 0.0001] were increased in serum from the DF group (details are shown in Table 4).

**Table 4:** Changes of SCFA between control group and DF group in serum

SCFA [μg/mL, M (IQR)]	Control group (*n* = 81)	DF group (*n* = 81)	*p* value
AA	0.97 (2.31)	0.99 (4.42)	0.276
PA	0.15 (0.22)	0.13 (0.18)	0.112
BA	0.05 (0.04)	0.80 (1.65)	< 0.0001
IBA	0.02 (0.05)	0.03 (0.06)	0.002
VA	0.02 (0.04)	0.03 (0.04)	0.001
IVA	0.02 (0.01)	0.02 (0.02)	0.001
HA	0.07 (0.02)	0.11 (0.03)	< 0.0001

*SCFA* short chain fatty acids, *AA* acetic acid, *PA* propionic acid, *BA* butyric acid, *IBA* isobutyric acid, *VA* valeric acid, *IVA* isovaleric acid, *HA* hexanoic acid

### Correlation analysis of the gut microbiota, SCFA and clinical indices in the DF group

**Fig. 5:**
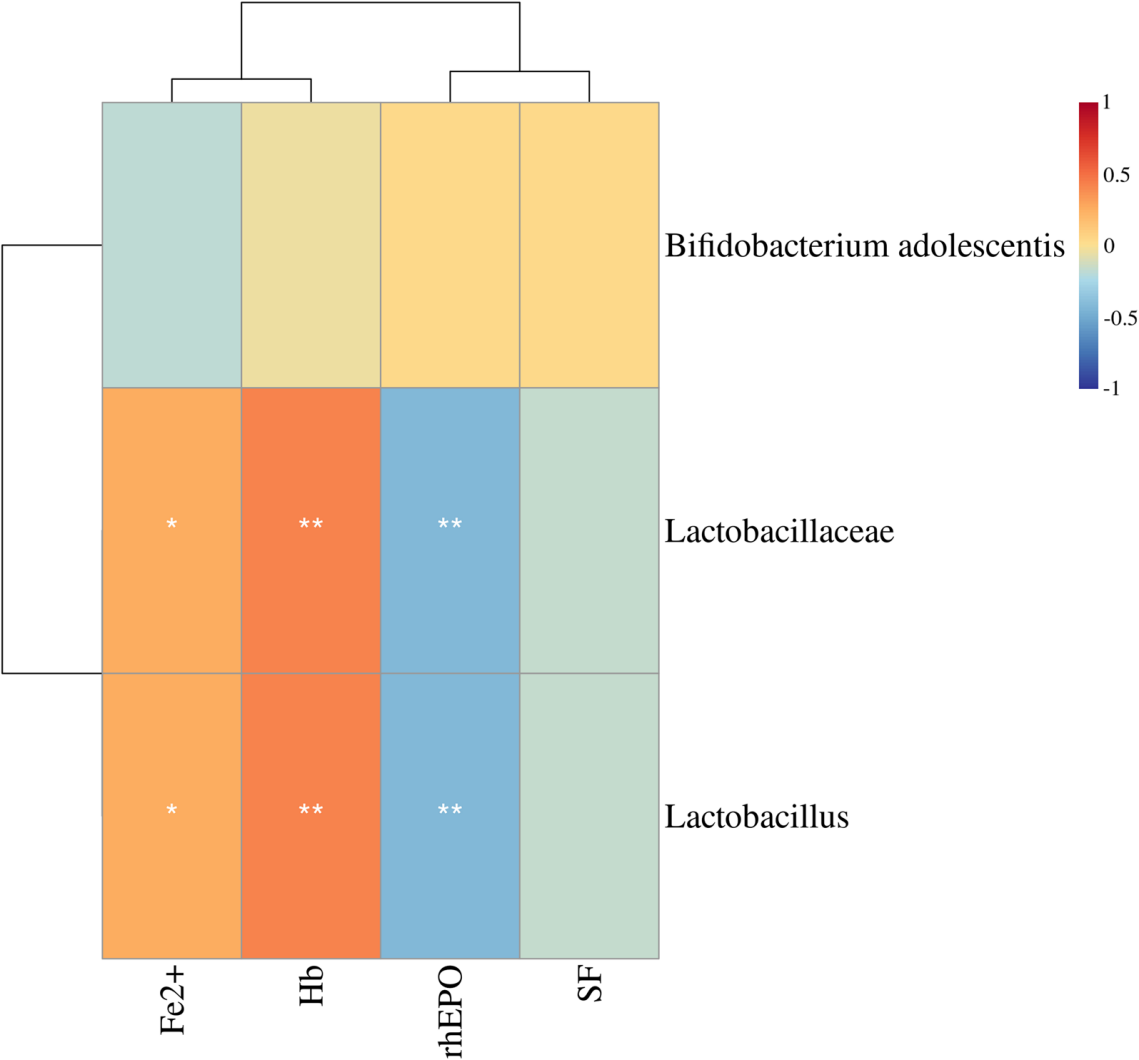
Heatmap of correlation coefficients between bacteria and clinical indicators in the DF group. *Lactobacillus* and *Lactobacillaceae* were positively correlated with Fe^2+^ and Hb and negatively correlated with rhEPO. *Hb* hemoglobin, *Fe*^*2*+^ serum ferrous iron, *SF* serum ferritin, *rhEPO* recombinant human erythropoietin. *: < 0.05, **: < 0.01. Correlation analysis was based on the Spearman correlation method

**Fig. 6:**
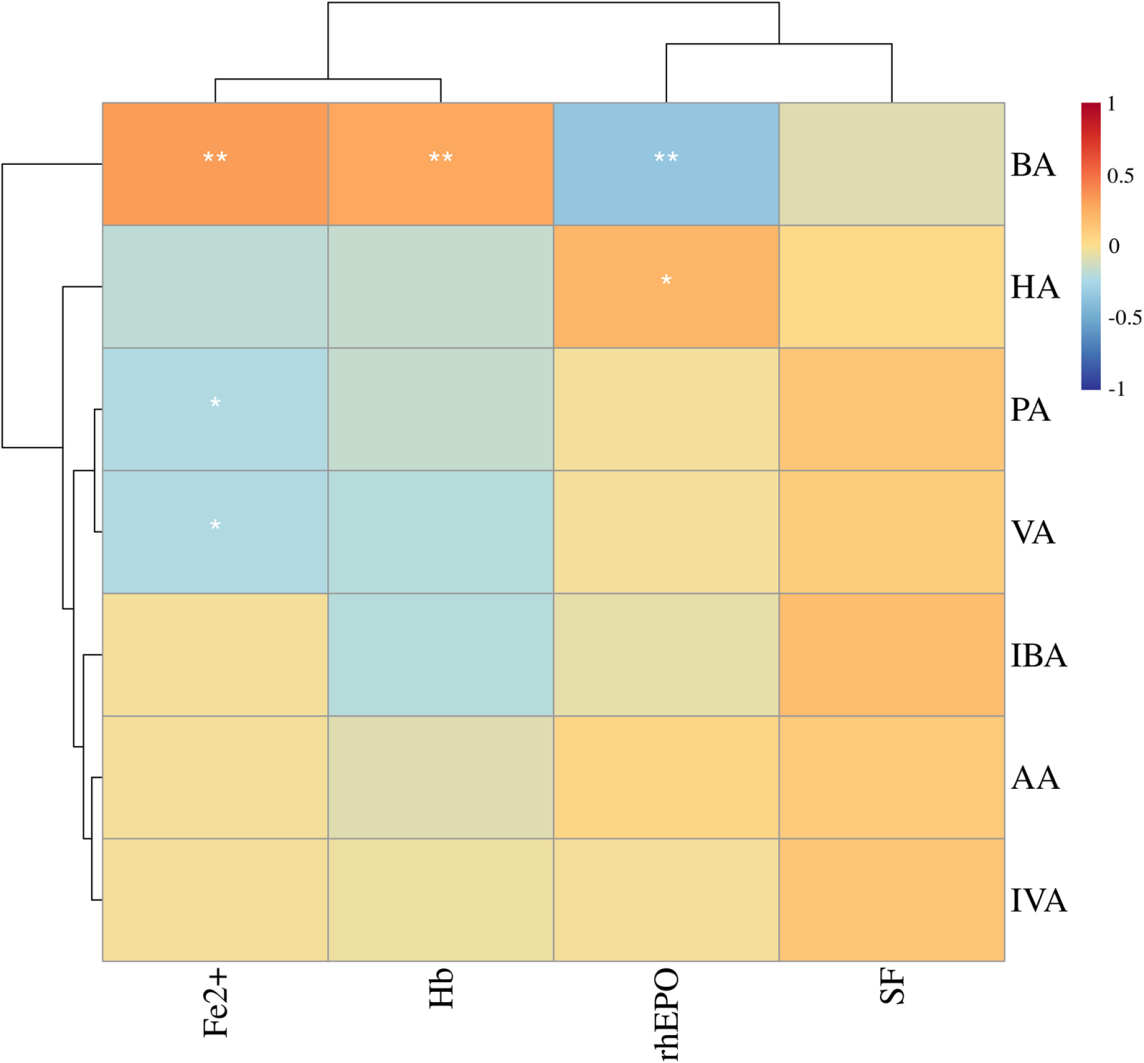
Heatmap of correlation coefficients between serum SCFA and clinical indicators in the DF group. BA was positively correlated with Fe^2+^ and Hb and negatively correlated with rhEPO. HA was positively correlated with rhEPO. PA and VA were negatively correlated with Fe^2+^. *Hb* hemoglobin, *Fe*^*2*+^ ferrous iron, *SF* serum ferritin, *rhEPO* recombinant human erythropoietin, *AA* acetic acid, *PA* propionic acid, *BA* butyric acid, *IBA* isobutyric acid, *VA* valeric acid, *IVA* isovaleric acid, *HA* hexanoic acid. *: < 0.05, **: < 0.01. Correlation analysis was based on the Spearman correlation method

**Fig. 7:**
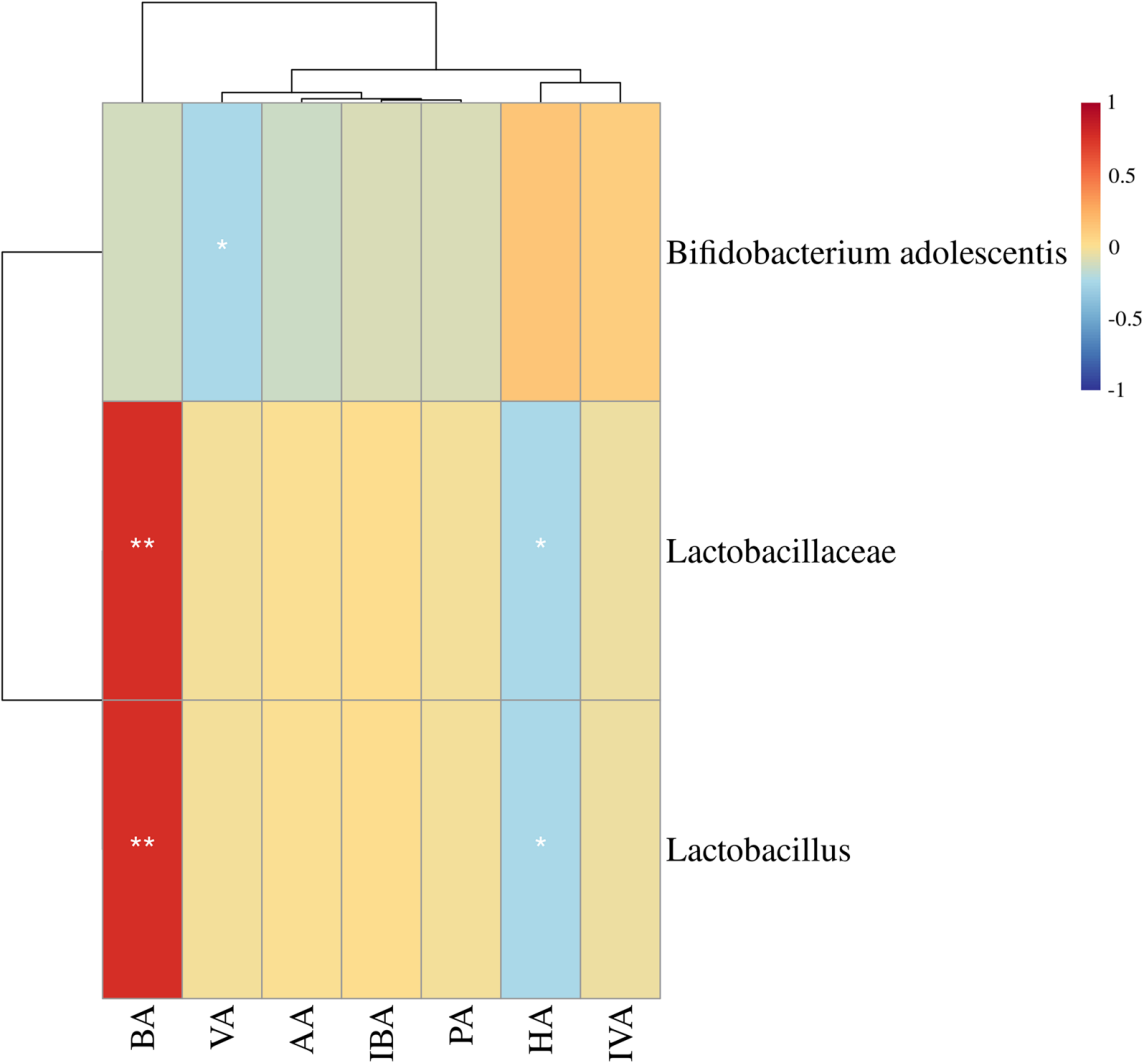
Heatmap of correlation coefficients between bacteria and SCFAs in the serum of the DF group. *Lactobacillus* and *Lactobacillaceae* were positively correlated with BA and negatively correlated with HA. *Bifidobacterium adolescentis* was negatively correlated with VA. *AA* acetic acid, *PA* propionic acid, *BA* butyric acid, *IBA* isobutyric acid, *VA* valeric acid, *IVA* isovaleric acid, *HA* hexanoic acid. *: < 0.05, **: < 0.01, ***: < 0.001. Correlation analysis was based on the Spearman correlation method

Spearman correlation tests for the gut microbiota and SCFA, gut microbiota and clinical indices, and SCFA and clinical indices in the DF group after intervention found that (1) the abundance of *Lactobacillus* and *Lactobacillaceae* were positively correlated with Hb (*r* = 0.44, *p* < 0.0001; *r* = 0.44, *p* < 0.0001) and Fe^2+^ (*r* = 0.44, *p* < 0.000; *r* = 0.44, *p* < 0.0001) (Fig. 5). (2) The serum BA level in the DF group was positively correlated with Hb (*r* = 0.29, *p* = 0.009) and Fe^2+^ (*r* = 0.32, *p* = 0.004) (Fig. 6). (3) The abundance of *Lactobacillus* and *Lactobacillaceae* in the DF group was positively correlated with the level of serum BA (*r* = 0.75, *p* < 0.0001; *r* = 0.75, *p* < 0.0001) (Fig. 7).

## Discussion

Renal anemia is a common and serious complication in ESRD patients and seriously threatens the safety of ESRD patients. Causes of renal anemia include decreased erythropoietin (EPO) production, iron dyshomeostasis, chronic inflammation, reduced red blood cell lifespan due to uremic toxins, and hematopoietic microenvironment disturbances, among others. With the application of rhEPO in the clinic, renal anemia has been effectively treated. However, an increasing number of patients also present clinically with EPO hyporesponse, which may be related to factors such as iron metabolism disorder and EPO receptor dysfunction. Recently, with the application of hypoxia inducible factor prolyl hydroxylase inhibitor (HIF-PHI) in the clinic, the standard rate of treatment of renal anemia in ESRD dialysis patients has been further improved [23, 24]. However, renal anemia is a multifactorial disease, and the diversity of its etiologies has contributed to its need for multitarget therapies.

Through this prospective, randomized, placebo-controlled clinical study, we showed that sufficient DF uptake improved Hb levels in patients with renal anemia. This finding is similar to the results reported in some previous animal studies and clinical studies [3, 25–28]. Moreover, our results showed that DF consumption notably elevated Hb levels in patients with renal anemia, with an average increase above 20%. This means that for renal anemia patients with a severe deficiency in DF intake, increasing DF consumption could have considerable therapeutic efficacy. In addition, we found that DF could change the gut microbiota structure of patients with renal anemia. For example, the anundance of *Bifidobacterium adolescentis*, *Lactobacillus*, and *Lactobacillaceae,* were increased, and the beneficial effects of *Bifidobacterium adolescentis*, *Lactobacillus*, and *Lactobacillaceae* on CKD patients have been demonstrated [29, 30]. Moreover, our study also found that oral DF increased serum SCFA (especially BA) concentrations in patients with renal anemia. *Bifidobacterium adolescentis*, *Lactobacillus*, and *Lactobacillaceae* are well-established as BA-production-related bacteria, and our results suggest that the effect of DF elevating Hb in patients with renal anemia may be associated with an increase in BA and BA-production-related bacteria [31–33]. BA is one of the most widely studied SCFAs and has been shown to exist in multiple mechanisms affecting anemia-related diseases. A study by Reid et al. found that BA derivatives ameliorated sickle cell anemia [17]. Weinberg et al. found that BA can promote the synthesis of γ-globin to improve thalassemia [18]. However, as renal anemia is a disease with multiple causes, it is unclear whether BA can improve this condition. Our study confirmed for the first time that BA produced by DF fermented by gut microbiota can improve renal anemia in a clinical trial. Correspondingly, we found that after DF intervention, the abundance of some potential pathogenic bacteria decreased. These species included *Acinetobacter* and *Ruminococcaceae*, which are related to infection, hemolysis and anorexia nervosa [34, 35]. However, we found that the relative abundance of Blautia, which is a BA-producing bacterium, was decreased in the DF group, but the serum BA level was elevated. It seems that there is a contradiction. We speculate that this may be related to the ability of *Bifidobacterium adolescentis, Lactobacillus*, and *Lactobacillaceae* to enhance butyrate production by other butyrate-producing bacteria. The specific mechanism needs further study.

Most of the current studies on the improvement of anemia by DF have focused on iron deficiency anemia. Carvalho and Freitas simultaneously reported that partially hydrolyzed guar gum was able to ameliorate iron deficiency anemia by improving iron absorption and iron metabolism status [3, 27]. Increased Fe^2+^ and SF levels in renal anemia patients after oral administration of DF were also found in our study, but the levels of sTfR were not notably affected. DF also had no obvious effect on hepcidin (an iron metabolism-related regulatory peptide). This is consistent with the findings of a recent systematic review that stated that DF has heterogeneous effects in ameliorating anemia by improving iron metabolism [5]. However, as stated in this systematic review, given the complexity of body iron metabolism and the variability in the design of individual studies, we cannot ignore the role of DF in improving iron metabolism and, in particular, its function of modulating gut bacterial biomarkers [5]. Our experimental data implicate the relevance of DF, the gut microbiota, SCFA, and iron metabolism, and our future research will investigate these associations.

As reported by Torres et al., BA stimulated Chinese hamster ovary cells to synthesize EPO in vitro, and BA was an important metabolite of DF and one of the most changed metabolites in our study [36]. Our study did not find a significant decrease in EPO consumption, although such a trend was indecated. As mentioned earlier, this trend could also be the result of improved iron metabolism or the effect of DF and its metabolites on erythropoietin receptor (EPOR) [37, 38]. However, whether DF can affect the synthesis of EPO in CKD patients has not been reported. Our research shows the potential function of DF in improving renal anemia by regulating EPO and EPO-related pathways, which is worthy of further study.

SCFAs have the potential to regulate bone marrow hematopoiesis. G protein coupled receptors (GPCRs) are the targets of SCFA. Trompette et al. found that propionate has the ability to regulate the differentiation of bone marrow hematopoietic cells by activating GPCR41, which mainly manifested as an increase in bone marrow-derived dendritic cells [22]. Accordingly, Docampo et al. found that butyrate can maintain the immune function of bone marrow-derived T cells by activating GPCR109A [39]. However, whether SCFAs can induce the differentiation of bone marrow erythroid hematopoietic cells by GPCRs needs further study.

Although our study raises the possibility that DF, the gut microbiota, and SCFA may improve renal anemia through the iron metabolism pathway or EPO-related pathway, there are still some limitations in this study. First, although the role of DF in health has been continually demonstrated, whether differences exist for different DFs has not confirmed by our study. We chose one mixture of DF, but whether other kinds of DF have similar or even better effects requires further research. Second, our study did not evaluate the long-term effects of DF on the gut microbiota. For example, whether the gut microbiota would be restored to its deleterious status after patients stopped using DF supplements was not investigated. How to use DF supplements to maintain the stability of the gut microbiota to achieve long-term therapeutic effects may be an interesting direction for future research. Third, our study did not clarify the exact mechanism by which DF improves renal anemia, but it provides a possible direction for our next basic research.

## Conclusion

DF ameliorates renal anemia by modulating the prebiotic activity of the gut microbiota and SCFAs and this effect warrants in-depth exploration in larger cohorts.

## Supplementary Information

**Additional file 1. **Material and Method Description of 16S rDNA Sequencing.

**Additional file 2.** Differences in bacterial composition between the two groups at the genus level.

## Data Availability

The datasets generated and analyzed in the current study are classified temporarily, as some of them may be used in our ongoing experiments. However, they might be provided by the corresponding author upon reasonable request.

## Abbreviations

ESRD: End-stage renal disease
DF: Dietary fiber
SCFA: Short-chain fatty acids
Hb: Hemoglobin
Fe^2+^: Serum iron
SF: Serum ferritin
sTfR: Soluble transferrin receptor
rhEPO: Recombinant human erythropoietin
BA: Butyric acid
EPO: Erythropoietin
EPOR: Erythropoietin receptor
eGFR: Estimated glomerular filtration rate
