# Supplementary material for: The prebiotic effects of soluble dietary fiber mixture on renal anemia and the gut microbiota in end-stage renal disease patients on maintenance hemodialysis: a prospective, randomized, placebo-controlled study
Source: J Transl Med. 2022 Dec 14;20:599. doi: 10.1186/s12967-022-03812-x (PMC9753397; doi:10.1186/s12967-022-03812-x)
Supplement: Supplementary file 1 — Additional file 1. Material and Method Description of 16S rDNA Sequencing. [file 12967_2022_3812_MOESM1_ESM.docx]

Material and Method Description of 16S rDNA Sequencing

1.DNA extractions

DNA from different samples was extracted using the E.Z.N.A.^®^ Stool DNA Kit (D4015, Omega, Inc., USA) according to manufacturer ’s instructions. The reagent which was designed to uncover DNA from trace amounts of sample has been shown to be effective for the preparation of DNA of most bacteria. Nuclear-free water was used for blank. The total DNA was eluted in 50 μ L of Elution buffer and storedat -80 °C until measurement in the PCR by LC-Bio Technology Co., Ltd, Hang Zhou, Zhejiang Province, China.

2.PCR amplification and 16S rDNA sequencing

| Region | Primers |
| --- | --- |
| V3-V4 | 341F (5'-CCTACGGGNGGCWGCAG-3')  805R (5'-GACTACHVGGGTATCTAATCC-3') |

The 5' ends of the primers were tagged with specific barcods per sample and sequencing universal primers. PCR amplification was performed in a total volume of 25 μL reaction mixture containing 25 ng of template DNA, 12.5 μL PCR Premix, 2.5 μL of each primer, and PCR-grade water to adjust the volume. The PCR conditions to amplify the prokaryotic 16S fragments consisted of an initial denaturation at 98 ℃ for 30 seconds; 32cycles of denaturation at 98 ℃ for 10 seconds, annealing at 54℃ for 30 seconds, and extension at 72 ℃ for 45 seconds; and then final extension at 72 ℃ for 10 minutes. The PCR products were confirmed with 2% agarose gel electrophoresis. Throughout the DNA extraction process, ultrapure water, instead of a sample solution, was used to exclude the possibility of false-positive PCR results as a negative control. The PCR products were purifyied by AMPure XT beads (Beckman Coulter Genomics, Danvers, MA, USA) and quantified by Qubit(Invitrogen, USA). The amplicon pools were prepared for sequencing and the size and quantity of the amplicon library were assessed on Agilent 2100 Bioanalyzer (Agilent, USA) and with the Library Quantification Kit for Illumina(Kapa Biosciences, Woburn, MA, USA), respectively. The libraries were sequenced on NovaSeq PE250 platform.

3.Data analysis

Samples were sequenced on an Illumina NovaSeq platform according to the manufacturer's recommendations, provided by LC-Bio. Paired-end reads was assigned to samples based on their unique barcode and truncated by cutting off the barcode and primer sequence. Paired-end reads were merged using FLASH. Quality filtering on the raw reads were performed under specific filtering conditions to obtain the high-quality clean tags according to the fqtrim(v0.94). Chimeric sequences were filtered using Vsearch software(v2.3.4). After dereplication using DADA2, we obtained feature table and feature sequence. Alpha diversity and beta diversity were calculated by normalized to the same sequences randomly. Then according to SILVA(release 132) classifier, feature abundance was normalized using relative abundance of each sample. Alpha diversity is applied in analyzing complexity of species diversity for a sample through 5 indices, including Chao1, Observed species, Goods coverage, Shannon, Simpson, and all this indices in our samples were calculated with QIIME2. Beta diversity were calculated by QIIME2, the graphs were drew by R package. Blast was used for sequence alignment, and the feature sequences were annotated with SILVA database for each representative sequence. Other diagrams were implemented using the R package(v3.5.2).
