# Supplementary figures and images for: The prebiotic effects of soluble dietary fiber mixture on renal anemia and the gut microbiota in end-stage renal disease patients on maintenance hemodialysis: a prospective, randomized, placebo-controlled study
Source: J Transl Med. 2022 Dec 14;20:599. doi: 10.1186/s12967-022-03812-x (PMC9753397; doi:10.1186/s12967-022-03812-x)

# Genus

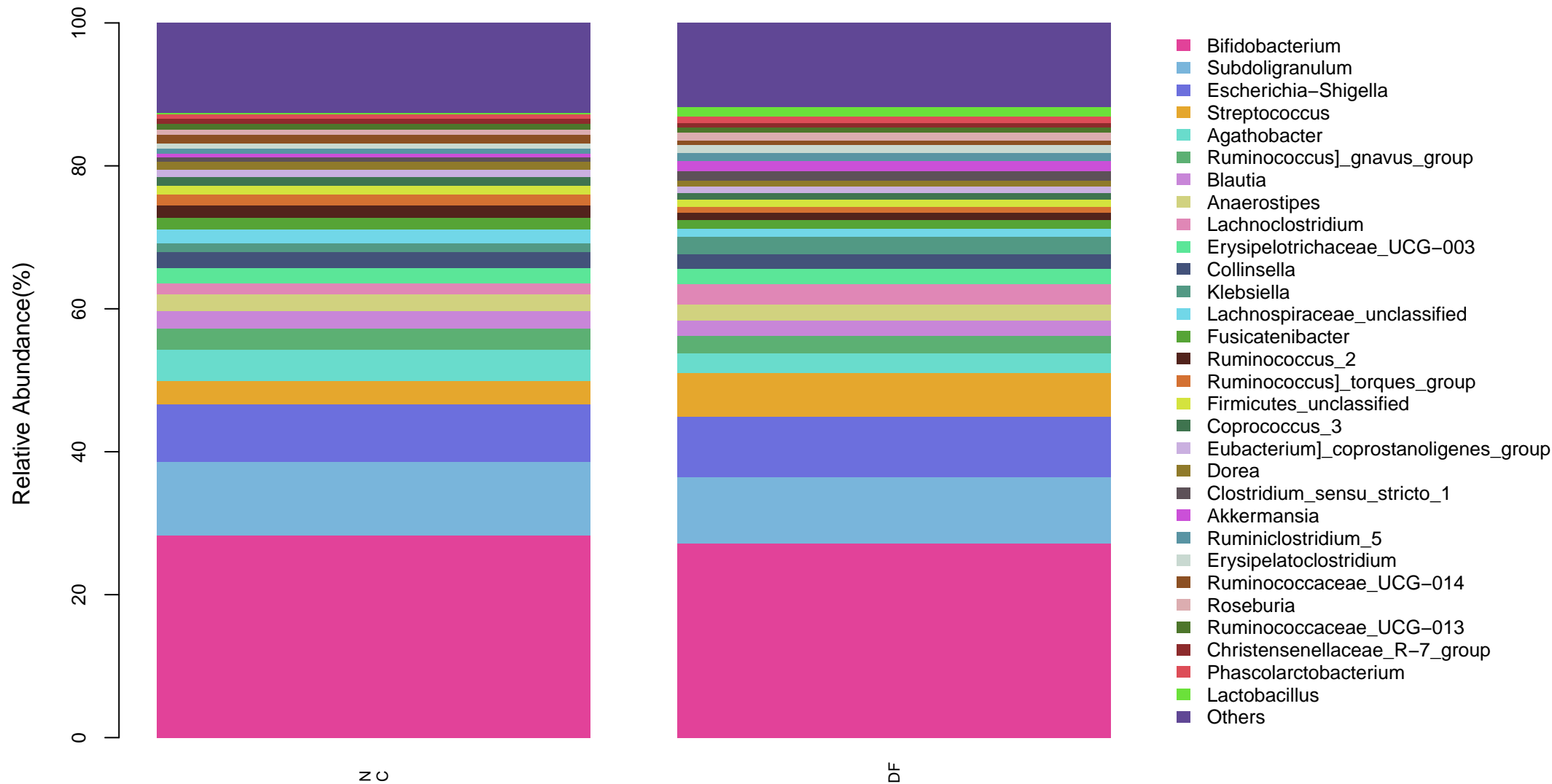

Supplement: Supplementary file 2 — Additional file 2. Differences in bacterial composition between the two groups at the genus level. [file 12967_2022_3812_MOESM2_ESM.pdf]
